# Stakeholders’ discourse on generative AI in higher education: Insights from topic modeling and sentiment analysis

**DOI:** 10.1371/journal.pone.0353685

**Published:** 2026-07-16

**Authors:** Wondwesen Tafesse, Mary Precy Aguilar, Sabaa Sayed, Maqsood Ahmad Sandhu

**Affiliations:** 1 Marketing and Entrepreneurship, United Arab Emirates University, Al Ain, United Arab Emirates; 2 College of Business Administration, University of Kalba, Sharjah, United Arab Emirates; Kazan University, RUSSIAN FEDERATION

## Abstract

Owing to their remarkable proficiency in academic tasks, generative AI (GenAI) systems have been widely embraced in the education sector, stimulating debate among academics about their opportunities and challenges. However, extant studies tend to be narrowly focused, typically drawing on micro-level observations from small samples of students and educators, which limits the scope of reported findings. The present study contributes to the literature by capturing stakeholders’ discussions on the role of GenAI in higher education at scale. It does so by employing over 230K tweets shared on the subject of GenAI and education and training a topic modeling algorithm on this corpus. The model identified seven major themes that encapsulate the use cases and implications of GenAI adoption in higher education as articulated by stakeholders: *learning assistant, research tool, productivity tool, assessment and examination, educational technology, adoption policy,* and *resources*. Additionally, the study performed sentiment analysis to reveal the specific themes on which stakeholders expressed disproportionately positive and disproportionately negative attitudes toward GenAI adoption. By combining stakeholder theory and a big data approach, the current findings highlight both the practical and institutional concerns of GenAI beyond immediate classroom implications, such as student learning and assessment.

## 1. Introduction

The remarkable capabilities of generative AI (GenAI) systems have spurred scholarly debate about their implications for higher education. On the one hand, the literature recognizes the enormous opportunities presented by GenAI, including enhancing personalized learning, reducing workload for educators and students, streamlining assessment and feedback, and facilitating on-demand access to educational content [1–3]. On the other hand, it recognizes several challenges, including the absence of quality assurance mechanisms [4], eroding students’ higher-order cognitive skills [5], and endangering academic integrity [6]. Discussions in the literature reflect both optimism about the potential benefits of GenAI and anxiety about its challenges [5].

Despite highlighting the optimism and anxiety about the adoption of GenAI in higher education, the extant literature falls short of considering the voices of influential stakeholders beyond students and educators. Important entities that have a stake in the adoption of GenAI in higher education, such as administrators, policymakers, and technology providers, are ignored [6]. Similarly, most existing studies lack an international perspective as their samples are mostly drawn from a few educational institutions typically located in a single country. As GenAI adoption expands globally, research that seeks to leverage insights from diverse international contexts is warranted [7].

To address some of the broader implications of GenAI in higher education, the current study draws on stakeholder theory, which, when applied in the education sector, calls for considering diverse perspectives from multiple stakeholders to devise and implement education policies [8]. Stakeholder involvement increases the quality of policy-making while also fostering policy acceptance through stakeholder engagement [9]. To acquire the perspectives of multiple stakeholders, this study employed a big data approach, leveraging a large corpus of tweets shared on the subject of GenAI and education. The tweet corpus provided access to the viewpoints of over 230K individuals based in various countries such as USA, UK, India, Canada, Australia, and Spain, among others. Despite capturing diverse viewpoints at scale, the tweet corpus did not specify the stakeholder position of individuals as students, academics, administrators, and so on. We inferred these stakeholder positions using a confidence interval approach based on a small subset of the tweet corpus. As this inference was carried out on a small subset of the tweet corpus, however, it did not allow for differentiation and analysis of stakeholder positions. This caveat notwithstanding, its sheer scale makes the data a vital source of stakeholder voices at scale.

The tweet corpus was analyzed using topic modeling—a natural language processing technique used to reduce large unstructured text data into concise and interpretable latent topics [10]. The topic modeling algorithm generated seven latent topics, which captured major concerns, opportunities, and use cases of GenAI as articulated by stakeholders. In addition, sentiment analysis was performed to demonstrate how stakeholders’ attitudes differ across the identified themes. This analysis revealed themes that garnered disproportionately positive and disproportionately negative sentiment from stakeholders. These analyses helped us answer the following two research questions:

1. *How are the implications of GenAI adoption in higher education articulated in stakeholders’ social media discourse?*
2. *Do sentiments differ across the thematic concerns articulated in stakeholders’ social media discourse?*

The findings contribute to the literature by leveraging the “wisdom of the crowd”. They combine stakeholder theory and a big data approach to present a macro view on the role of GenAI in higher education, thereby complementing the micro view offered in the literature. While the micro view emphasizes immediate classroom concerns such as student learning and assessment, heavily relying on the opinions of students and educators, the macro view goes beyond these immediate classroom concerns to surface broader issues of interest to stakeholders, including GenAI literacy, responsible adoption policy, academic integrity, and supporting ecosystem. Our findings, thus, surface the practical (e.g., learning, research) as well as institutional (resources, adoption policy, ecosystem) concerns of GenAI. The findings further contribute to the literature by demonstrating how large-scale social media discourse can be leveraged for early insight into disruptive technologies. While the data analyzed here captures stakeholders’ opinions during the early days of ChatGPT, it was able to identify issues that later became prominent, such as academic integrity and GenAI literacy.

## 2. GenAI in higher education

The impressive capabilities of GenAI models have spurred a significant body of scholarly debate regarding their implications for higher education. The literature broadly recognizes the transformative potential of GenAI models for teaching and learning, while simultaneously acknowledging the numerous challenges and risks they pose. The review presented in this section summarizes the key tensions in the literature to establish the context for our subsequent analysis.

Studies emphasizing the positive impact of GenAI cite the technology’s potential for personalized learning, creativity, and resource efficiency when integrated into teaching and instruction. For instance, Rospigliosi [11] argues that GenAI fosters personalized learning by enhancing the appropriability (ability to make knowledge one’s own), evocativeness (ability to provoke personal reflection), and integration of knowledge among students (ability to combine new knowledge with existing one). Jeon and Lee [12] highlighted how GenAI and human instructors can complement each other to foster personalized learning environments. The researchers identified three complementary instructor roles: orchestrating the resources afforded by GenAI, guiding students on the use of GenAI, and raising ethical awareness about GenAI; and four complementary roles of GenAI: interlocutor (role player), content provider (synthesizing instruction material), teaching assistant, and evaluator (assisting in assessment).

GenAI further enables both educators and students to brainstorm ideas, work out solutions, and develop content with ease [7,13], thereby infusing creativity into instruction and learning. For instance, Chan and Hu [14] reported that students perceive GenAI to be beneficial for obtaining immediate feedback, brainstorming ideas, and receiving support for research and analysis. Yan [15] reported that students perceive GenAI to be beneficial in terms of receiving quick feedback, regenerating responses, and developing solutions through incremental interactions. Other opportunities identified across various studies include personalizing content and tailoring the pace of learning to individual student needs [14,16]; and providing real-time feedback and assistance, which saves time and resources for both students and instructors [1,13].

This optimistic perspective is countered by significant concerns regarding the systematic risks and limitations posed by GenAI. Farrokhnia et al. [4] employed a SWOT analysis to outline the strengths, weaknesses, opportunities, and threats of GenAI in higher education. While recognizing strengths like personalization and real-time responses, the study identified critical weaknesses and threats related to GenAI, including the absence of quality checks, the risk of biases and discrimination, and the potential for weakening students’ higher-order cognitive skills due to over-reliance. These findings epitomize the inherent contradiction of GenAI, whereby a technology praised for enhancing student learning is simultaneously feared for undermining critical thinking and problem-solving skills [2]. The most pervasive challenge identified across studies is the threat to academic honesty. When used as an answer generator, rather than an intelligent tutor that walks students through learning material, GenAI inadvertently encourages malpractices such as plagiarism and cheating [5,17]. Further concerns related to GenAI’s lack of deep understanding, its inability to provide supporting evidence, and the risk of perpetuating existing biases embedded in its training data increase the risk of fully embracing GenAI in higher education [4].

Despite presenting a detailed discussion of the opportunities and challenges of GenAI in higher education, some of the studies reviewed in this section rely on conceptual synthesis and hence lack a solid empirical basis to support their claims. When data is used, the scope is typically limited to a small sample of students and educators drawn from a single higher education institution, and identified issues tend to gravitate toward immediate classroom concerns such as student learning and assessment. This narrow scope fails to capture broader implications of GenAI in higher education, such as institutional concerns related to GenAI adoption, the emergence of a GenAI ecosystem, or the role of GenAI literacy and capacity building for a GenAI environment. The present study addressed these limitations by analyzing large-scale social media discourse, which is ideal for surfacing institutional and practical issues of interest to stakeholders.

## 3. Stakeholder theory and big data analysis: A synergistic approach

Stakeholder theory is a theory of organizational management and ethics [18], with the core premise that organizations should create value for all their stakeholders to ensure their long-term success [19]. Stakeholders are defined as any group or individual who can affect or is affected by the achievement of an organization’s objectives [20]. The theory emphasizes that organizations are embedded in a web of relationships with diverse stakeholders, including employees, clients, suppliers, communities, and regulators, and managing these relationships is central to their long-term success [21]. The theory prioritizes inclusion and transparency by advocating for a fair and participatory decision-making process that incorporates diverse voices [20].

In the higher education context, stakeholder theory is especially pertinent given their public mission and complex stakeholder landscape, consisting of students, faculty, policy makers, industry partners, alumni, and local communities [9]. Higher education institutions are expected to balance societal expectations with resource efficiency, making stakeholder engagement vital to attaining this balance [8,22]. Engaging stakeholders improves the ability of higher education institutions to scan the environment, gather diverse external inputs, and make better decisions [9,23]. Stakeholder theory provides a suitable framework to navigate a dynamically changing environment characterized by continued pressure from external factors including technology, politics, and society [8].

A big data approach provides a sound empirical basis for applying stakeholder theory [24]. The volume, variety, and velocity of big data, especially those mined from social media, enable learning about the perspectives of diverse stakeholder groups on matters related to education policies or technological disruptions [25,26]. Big social media data allows observing broader patterns and relationships in real time, by leveraging information shared on social media by different stakeholders [27]. Students, teachers, parents, administrators, and consultants use social media for communication, which creates a vast trove of high-frequency, real-time data that can offer valuable insight for decision-making [6]. Wang [26] aptly noted the value of big social media data for education policy-making as follows:

“In addition to participating in periodic polls and surveys, Internet users, in particular social media users and bloggers, frequently share their opinion and sentiment online. These online comments and messages, as the new sources of public opinion, are of great value for policymakers to understand problems and public needs, formulate policies to address them, evaluate policy effectiveness, and even engage the public in idea generation and problem solving”.

Other researchers reinforce the value of big social media for generating unique insight. For instance, Chen et al. [28] argued that big social media data is ideal to understand students’ emotional and subjective learning experiences as they use social media to informally converse about their school experiences.

Building on the preceding insights, this study analyzes a large volume of personal opinions shared on X (formerly Twitter) by different stakeholders on the subject of GenAI and education. X is a major social media platform used by millions of users worldwide to share their views in real-time [29]. More than 500 million tweets are posted on the platform daily [30]. This extensive data stream provides a valuable and varied resource of information about what stakeholders think about GenAI and its role in higher education [31].

However, our approach to marry stakeholder theory with big social media data is not without limitations. Specifically, due to the pre-anonymization of the dataset used, we were not able to assign each tweet to a specific stakeholder group, such as student, academic, or administrator. This, in turn, made it difficult to dissect the social media discourse across different stakeholder groups. As a result, the application of stakeholder theory in this study was confined to uncovering generic stakeholder discourse without being able to analyze potential differences across stakeholder groups.

## 4. Methodology

### 4.1. The dataset

**Fig 1:**
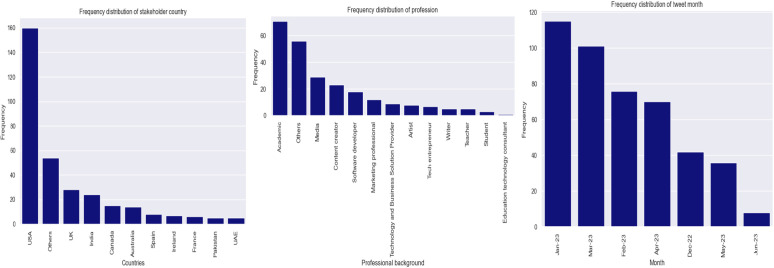
Frequency distribution of the metadata.

The dataset used in this study was obtained from kaggle.com, an open access platform where data scientists publicly share data and collaborate on projects. The dataset consisted of 233,000 tweets about ChatGPT and education. Despite its scale, the dataset lacked detailed metadata (e.g., specific tweet date, user profile, and user location), without which the planned stakeholder analysis could not be performed. Given the scale of the dataset, manually collecting the metadata for each tweet was unrealistic. A more practical approach was to take a random sample of tweets from the original dataset and manually gather the metadata to make inferences about the entire tweet corpus using a confidence interval approach [32]. This approach allows learning about the sources of the tweets without collecting data from each tweet. With this in mind, a random sample of 500 tweets was selected from the tweet corpus, and the metadata was manually gathered from X. The information collected included tweet dates, user profile, and country of residence. The frequency distributions of these variables are plotted in Fig 1.

As the metadata variables were all categorical observations with multiple categories, the confidence interval approach for multinomial proportions was employed to make inferences, which involves estimating a range of proportions that contain the true (population) proportions [33]. In our case, the lower and upper bounds for the proportion of each category were estimated with a 95% confidence level. The confidence interval estimates were computed using the Goodman method, which applies a chi-square distribution to calculate the upper and lower bounds using the number of observations in each category and the total sample size as inputs [33]. The results of the confidence interval estimates are reported in Table 1.

**Table 1:** Confidence interval for multinomial proportions.

Categorical variables	Sample proportion	95% confidence interval	
Lower bound	Upper bound
User country			
*USA*	32.2%	28.2%	36.6%
*Others*	10.9%	8.2%	13.9%
*UK*	5.6%	3.8%	8.1%
*India*	4.8%	3.1%	7.1%
*Canada*	3%	1.7%	4.9%
*Australia*	2.8%	1.6%	4.7%
*Spain*	1.6%	0.7%	3.1%
*Ireland*	1.4%	0.6%	2.9%
*France*	1.2%	0.4%	2.6%
*Pakistan*	1%	0.3%	2.3%
*UAE*	1%	0.3%	2.3%
User profession			
*Academic (university professor, lecturer, researchers)*	14.3%	11.3%	17.7%
*Others*	11.3%	8.6%	14.4%
*Media*	5.8%	3.9%	8.3%
*Content creator*	4.6%	2.9%	6.9%
*Software developer*	3.6%	2.1%	5.7%
*Marketing professional*	2.4%	1.3%	4.2%
*Technology and business solution provider*	1.8%	0.8%	3.4%
*Artist*	1.6%	0.7%	3.1%
*Technology entrepreneur*	1.4%	0.6%	2.9%
*Writer*	1%	0.3%	2.3%
*Teacher*	1%	0.3%	2.3%
*Student*	0.6%	0.1%	1.8%
*Education technology consultant*	0.2%	0.05%	1.1%
Tweet month			
*January 2023*	23.2%	19.5%	27.1%
*March 2023*	20.3%	16.9%	24.1%
*February 2023*	15.3%	12.2%	18.8%
*April 2023*	14.1%	11.1%	17.5%
*December 2022*	8.5%	6.2%	11.3%
*May 2023*	7.2%	5.1%	9.9%
*June 2023*	1.6%	0.7%	3.1%

Most stakeholders are based in the USA, followed by those based in the UK, India, and Canada. The “Others” category is an aggregate of countries with fewer than five observations. In terms of profession, academics (university professors, lecturers, and researchers), media organizations (traditional media, websites, blogs, etc.), content creators, and software developers have greater representations in the dataset. The “Others” category is an aggregate of professions with fewer than five observations. Finally, in terms of the tweet publication date, most of the tweets were shared in January 2023, followed by March and February 2023. Most of the tweets were, thus, shared within the first three months of ChatGPT’s public release on November 30, 2022. Although this data might be considered outdated given the rapid pace of GenAI development, it offers a unique window into stakeholders’ initial reactions to the technology.

With large-scale social media data, ethical handling of data is a critical concern. In this study, we ensured the data was acquired ethically from an open access data repository platform. As the original dataset was pre-anonymized, the identity of the tweet authors remained anonymous throughout the data analysis, which ensured user privacy and confidentiality. Our analytical approach further enhanced the contextual integrity of the data by focusing on mining aggregated opinions while avoiding individual scrutiny of tweet authors.

Finally, we would like to point out the limitations of the dataset used, especially as it pertains to supporting a deeper exploration of stakeholder theory. First, the dataset captured early social media discourse at scale, but it is limited in representing stakeholder groups across geographies and roles. The way social media data is created and disseminated is inherently biased, favoring specific geographies and roles, while under-privileging others. In our case, for instance, stakeholders from non-Western countries and those related to students and university administrators were underrepresented. Second, the pre-anonymization of the dataset precluded a meaningful differentiation of attitudes, motivations, and interests across stakeholder positions, which is of central importance to stakeholder theory. For instance, we could not establish whether certain stakeholder groups exhibited greater interest in specific GenAI adoption themes. Due to these limitations, stakeholder theory could not be fully explored, especially as it pertains to differences in interests and motivations across stakeholder groups.

### 4.2. Topic modeling

Topic modeling is a natural language processing technique aimed at uncovering latent themes within a large body of text [10]. It works by detecting patterns of words that frequently appear together and grouping them into topics [34]. Topic modeling is particularly effective for condensing a large text corpus into a set of underlying topics characterized by a collection of related words [35].

To implement topic modeling on the tweet corpus, Latent Dirichlet Allocation (LDA) was used, which is one of the most widely used topic modeling algorithms [34]. LDA is a generative probabilistic model, and its underlying tenet is that documents (such as tweets) are composed of multiple topics, and these topics are composed of related words [35]. Specifically, LDA views each document as a probabilistic mixture of topics, and each topic as a probabilistic distribution over the finite vocabulary of words found in the corpus [34]. The model assumes a hypothetical process where an author first chooses a mix of topics for a document and then selects words based on the probability distribution of words associated with those topics [10].

When applied to the tweet corpus, LDA’s output helps to assign each tweet to specific latent topics with certain probabilities. It also provides the word distribution over the latent topics, thereby revealing their underlying themes. In the next section, LDA’s implementation on the tweet corpus is discussed.

### 4.3. LDA implementation

Before training an LDA model, the input text must undergo preprocessing, which typically includes tokenization, removing stop words, and applying lemmatization [36]. Tokenization involves breaking the input text into smaller units called tokens, enabling LDA to analyze each of them individually. Stop words, which include prepositions, conjunctions, articles, and pronouns, as well as special characters such as punctuation marks, emoticons, links, and usernames, are less useful for topic modeling. Consequently, these elements were excluded from the tweets. Lemmatization converts words into their base form or lemma. For instance, words like “taking,” “takes,” and “took” are all converted to “take” through lemmatization, thereby reducing the number of unique terms in the text corpus. Lastly, high-frequency non-stop words were removed from the tweet corpus, as they are less valuable for topic modeling due to their ubiquity [36]. In this study, “chatgpt” and “education” were the most frequently occurring terms and, thus, were excluded.

**Fig 2:**
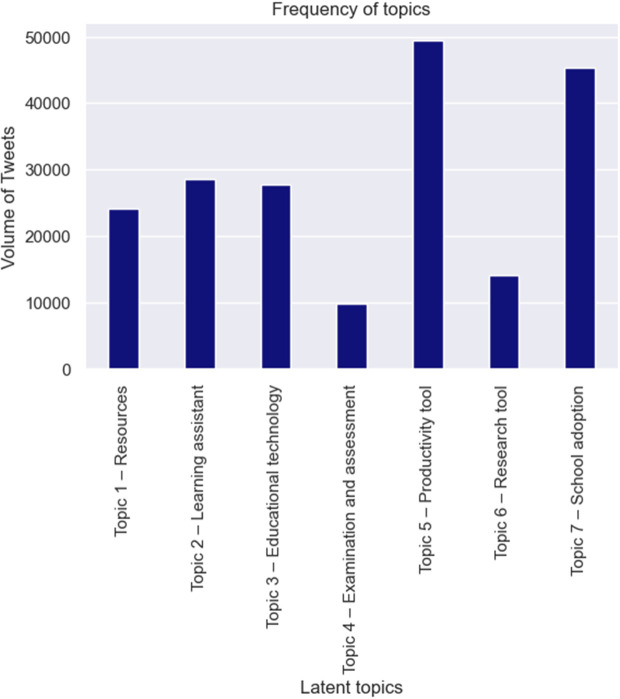
Volume of tweets assigned to the latent topics.

Subsequently, the LDA model was trained on the preprocessed tweet corpus. The LDA model requires specifying the number of topics beforehand, which makes determining the optimal number of topics an important but challenging task. Researchers typically do this by iterating through different topic numbers and assessing the validity of the resulting topic models [35,36]. In line with this approach, we iteratively trained the LDA model, starting with one topic and incrementally increasing to 20 topics. This iterative process facilitates the exploration of various potential topic structures within the text corpus, ultimately helping to identify the most robust and usable model. Eventually, the topic model with seven topics was selected as it produced a highly interpretable topic structure. Interpretability was judged based on the distinctiveness of the emerging topics. When a topic model produces distinct topics, it generates a more complete representation of the data, while also being easier to interpret due to a clean conceptual separation among the resulting topics. The seven-topic structure was deemed highly interpretable for this reason. The number of topics was neither too many nor too few. This balance allowed the topic structure to capture distinct themes that could be systematically interpreted through careful analysis of top-words and sample documents assigned to each topic with high probabilities. This can also be seen from the high coherence score achieved by this particular topic structure relative to other topic structures. Coherence score evaluates the similarity of the top words associated with each latent topic [37], with high scores indicating sharper distinction among the resulting topics. The frequency distribution of the latent topics is illustrated in Fig 2.

## 5. Results

### 5.1. Labeling the topics

The seven latent topics produced by the LDA were labeled based on the top ten words assigned to each topic with the highest probabilities. Furthermore, several representative tweets assigned to each latent topic with high probabilities were analyzed. The actual task of labeling and interpreting the topics were carried out by the authors in a collaborative group discussion. The top words and representative tweets generated by the topic model was shared among authors beforehand so that they can derive labels and core themes individually. During the group discussion session, each author presented their derived labels and themes. These were subsequently consolidated and refined into unified labels and themes through discussion and consensus. Subsequently, these unified labels and themes were converted into narrative text by the first author and shared with the other authors on a cloud-based file editing platform for a live, iterative refinement and development. The summary of the topics shared below is a result of this process. Table 2 summarizes the distribution of the top words and their probabilities used in labeling and interpreting the topics, while Table 3 provides a snapshot of representative tweets.

**Table 2:** Distribution of top ten words assigned to the latent topics.

Assigned Topic	Top-words	Probabilities
Topic 1 – Resources	Course	0.026
	Research	0.025
	GPT	0.022
	AI	0.018
	Use	0.017
	Prompt	0.013
	Free	0.01
	Content	0.01
	Create	0.008
Topic 2 – Learning assistant	Student	0.06
	Use	0.04
	AI	0.038
	Tool	0.02
	Learn	0.018
	Teacher	0.018
	Education	0.016
	Write	0.016
	University	0.012
	Cheat	0.01
Topic 3 – Educational technology	AI	0.06
	Education	0.04
	Research	0.02
	Technology	0.01
	New	0.01
	Intelligence	0.01
	Artificial	0.009
	OpenAI	0.008
	Future	0.008
	Learn	0.008
Topic 4 – Examination and assessment	Exam	0.08
	Pass	0.02
	Medical	0.019
	GPT	0.017
	Law	0.017
	Pas	0.015
	Bar	0.015
	School	0.01
	Professor	0.01
	Test	0.01
Topic 5 – Productivity tool	Research	0.016
	Use	0.014
	Think	0.013
	Ask	0.011
	GPT	0.01
	Like	0.01
	Write	0.01
	Question	0.009
	Answer	0.009
	Get	0.009
Topic 6 – Research tool	Research	0.018
	Use	0.017
	Student	0.009
	Language	0.009
	Write	0.008
	Paper	0.008
	Model	0.008
	GPT	0.007
	Ask	0.007
	Generate	0.007
Topic 7 – School adoption policy	Write	0.023
	School	0.021
	Use	0.018
	GPT	0.017
	Essay	0.017
	Chat	0.014
	Student	0.013
	Teach	0.012
	Get	0.011
	Go	0.011

**Table 3:** Sample tweets assigned to the latent topics.

Sample tweet	Latent topic	Probability	Sentiment
Want to boost your tutoring productivity and creativity? Our online course on ChatGPT prompts has got you covered!	Topic 1 – Resources	0.68	Neutral
Yes, students use technology to cheat, but they also use it to learn, and history has shown us repeatedly that far more students use it to learn than cheat.	Topic 2 – Learning assistant	0.78	Positive
Integration of new AI tools like GPT-4 and Mid Journey into nonformal education would be the next step in improving youth work and education in general	Topic 3 – ChatGPT as an educational technology	0.85	Positive
I agree with the purpose of essays, but I don’t think Chat GPT really threatens well-designed assignments or essays which truly require deep understanding. I’ve asked it about deep topics and received confidently wrong answers. I definitely wouldn’t submit an essay it wrote.	Topic 4 – examination and assessment	0.95	Negative
Using AI effectively requires us to engage our critical thinking and ask thoughtful questions. AI is a tool, and intelligently used tools are always more effective. A hammer can put a hole in a wall as effectively as it can drive a nail.	Topic 5 – Productivity tool	0.74	Positive
Finally got email about research ethics and using ChatGPT lmao. “ChatGPT cannot be listed as an author”	Topic 6 – Research tool	0.71	Positive
This level of AI is insane, to be on the receiving end of papers generated by ChatGPT is a horrible feeling, makes me feel like all the work I put into teaching is useless	Topic 7 – School adoption policy	0.95	Negative

#### 5.1.1. Topic 1 – Resources.

This topic is mainly about the resources available to effectively use GenAI in a higher education environment. The distribution of words assigned to Topic 1 with high probabilities indicates that the discourse in this topic emphasizes the resources available to learn ChatGPT for research, writing, and creative tasks. Examination of several sample tweets assigned to Topic 1 with high probabilities emphasizes the importance of acquiring GenAI skills related to prompting GenAI chatbots and validating GenAI outputs. The sample tweets discussed ChatGPT-related courses, tutorials, and tools designed to build GenAI skills. The discussion in this topic reflects the high demand for learning resources during the early days of ChatGPT, suggesting that public discourse was already organizing around capacity building for what stakeholders view as a future increasingly enabled by GenAI models. Given that this was an early dataset, the discovery of this topic suggests that stakeholders understood the value of GenAI literacy early on, a competency that today is widely recognized as essential for the effective and responsible GenAI adoption in higher education [38].

#### 5.1.2. Topic 2 – Learning assistant.

This topic is mainly about the role of GenAI as a learning assistant, highlighting the various ways students can use GenAI to support their learning goals. The distribution of words assigned to Topic 2 with high probabilities reflects stakeholders’ interest in how students are using ChatGPT to support their learning. Additionally, sample tweets assigned to Topic 2 with high probabilities point to the opportunities (e.g., personalized tutoring) and challenges (plagiarism, cognitive off-loading) of utilizing ChatGPT as a learning assistant. By simultaneously capturing the opportunities and risks of GenAI as a learning assistant, Topic 2 amplifies the tension between GenAI optimism and GenAI anxiety that permeates academic discussions [2,5]. The sub-themes within this topic reiterate stakeholders’ strong interest in promoting responsible GenAI adoption among students by balancing GenAI use with more independent, self-regulated learning [38].

#### 5.1.3. Topic 3 – Educational technology.

This topic framed GenAI as an emerging educational technology, highlighting its disruptive effects across teaching, learning, and research practices in higher education institutions. The distribution of top words assigned to Topic 3 with high probabilities indicates that this topic characterizes ChatGPT as a disruptive, fast-moving technology with immediate implications for higher education institutions. Sample tweets assigned to Topic 3 point to potential disruptions in assessment, instruction, and student learning stemming from ChatGPT’s technical capabilities and rapid adoption among students and faculty. Some of the tweets discuss the technology behind ChatGPT, the main players, and technical advances in the AI sector. Thus, discussions on this topic surface a confluence of technological and educational issues, where the technology’s capabilities and its ecosystem are framed in terms of their impact on student learning and pedagogical practices. This stakeholder discourse mirrors the view in the literature that a deeper understanding of the technology behind GenAI and its ecosystem is necessary for its thoughtful adoption in higher education [39,40].

#### 5.1.4. Topic 4 – Examination and assessment.

This topic discusses the implications of GenAI for examination and assessment practices in higher education. The distribution of top words assigned to Topic 4 with high probabilities indicates that the discourse in this topic is organized around how ChatGPT can disrupt traditional examination and assessment practices in higher education institutions. Additionally, sample tweets assigned to Topic 4 with high probabilities indicate stakeholders’ concerns about the widespread adoption of ChatGPT by students and its effect on the integrity and effectiveness of exams and assessments. Some of the tweets argued for the need to rethink traditional assessment tools that are vulnerable to GenAI adoption, such as home-based assignments and essays. Other tweets mentioned available tools and methods for screening GenAI-generated content. The discourse in Topic 4 captured what later became one of the most pressing policy concerns for higher education institutions―how to evolve assessment practices in response to the growing adoption of GenAI by students [3]. Discussions frequently question the adequacy of traditional assessment methods in light of the active use of GenAI tools for assignment completions.

#### 5.1.5. Topic 5 – Productivity tool.

This topic characterized GenAI as a productivity tool in the higher education context, discussing not only its productivity benefits but also its productivity pitfalls. The distribution of top words assigned to Topic 5 with high probabilities alludes to some of the major capabilities of ChatGPT as a productivity-enhancing tool, including its ability to answer questions, brainstorm ideas, and write documents. Sample tweets assigned to Topic 5 with high probabilities discuss various applications of ChatGPT that can improve productivity, including essay writing, finding answers, code writing, and completing creative and reasoning tasks. The tweets also highlight the pitfalls of ChatGPT in delivering quality responses, including hallucinations and providing inaccurate, misleading, or biased outputs. By pointing out both the productivity gains and pitfalls of GenAI, this topic reflects stakeholders’ dual view of the technology as a productivity tool. Notably, stakeholders’ discussion under this topic presciently surfaced a phenomenon that has since gained prominence—AI slop. It describes how GenAI output often falls short of users’ expectations, leaving them with the burden of reviewing and revising it, thereby diminishing rather than boosting productivity [41].

#### 5.1.6. Topic 6 – Research tool*.*

This topic discussed the value of GenAI as a research tool, highlighting the various ways students and researchers can use GenAI tools to support their research efforts. The distribution of top words assigned to Topic 6 with high probabilities indicates that the discourse in this topic revolves around ChatGPT’s capabilities as a research tool, including generating and brainstorming research ideas, asking questions, and drafting research content. Sample tweets assigned to Topic 6 with high probabilities discuss several research-related themes, such as ChatGPT’s research capabilities, ways to responsibly use the technology for research, policies related to the use of ChatGPT in research, and ChatGPT’s authorship of research papers. The discussion in this topic highlights stakeholders’ view of an important yet underexplored use case of GenAI in higher education: its role as a research tool. Compared to more widely discussed use cases of GenAI, such as learning assistance and instructional support, research-related applications garnered limited attention in the academic literature [42]. Moreover, in the sub-themes identified under this topic, stakeholders raise concerns about the use of GenAI in research, including research integrity, the reliability of using GenAI output in research, and the question of authorship status, which reinforce the limited discussions in the academic literature [1].

#### 5.1.7. Topic 7 – School adoption policy.

In this topic, stakeholders discussed policies instituted by higher education institutions to regulate GenAI adoption. The distribution of top words assigned to Topic 7 with high probabilities highlights school, teacher, and student reactions to actual cases of ChatGPT adoption in the classroom. The adoption of ChatGPT in schools and stakeholders’ responses to it constitute a unique theme of this topic. Sample tweets assigned to Topic 7 with high probabilities discuss school adoption policies, including well-publicized cases of schools imposing various forms of restrictions during the early days of ChatGPT. Thus, Topic 7 reflects an important and ongoing concern among higher education institutions about how to develop proactive policies to promote responsible and ethical adoption of GenAI [43]. Higher education institutions are struggling to formulate effective policy guidelines, as the technology profoundly disrupts established educational practices, from curriculum and instructional design to assessment practices, while also evolving at a breakneck speed [44]. Early stakeholders’ discourse surfaced these challenges in advance, drawing attention to instances of irresponsible adoption and hasty policy responses from higher education institutions.

### 5.2. Sentiment analysis

Sentiment analysis was performed to reveal stakeholders’ attitudes about the adoption of GenAI in higher education as positive, negative, or neutral. For this purpose, TextBlob was employed, a popular sentiment analyzer in Python. TextBlob has been applied in prior educational research to measure stakeholder sentiment [45]. It utilizes a vast lexicon of English adjectives and adverbs manually tagged with “polarity” scores to measure their emotional tone [46]. The polarity scores range between −1 (extreme negative emotion) and +1 (extreme positive emotion), with a polarity score of zero representing a neutral sentiment. For each document in a text corpus, TextBlob identifies the adjectives and adverbs using a part-of-speech tagger and retrieves their polarity scores. These individual scores are then aggregated to produce a single score per document ranging between −1 and +1. The polarity score is adjusted for the existence of modifying adverbs, negations, emojis, and punctuation marks [46,47]. For ease of interpretation, we coded the continuous polarity score for each tweet into three discrete levels: negative (polarity score < 0), neutral (polarity score = 0), and positive (polarity score > 0).

**Fig 3:**
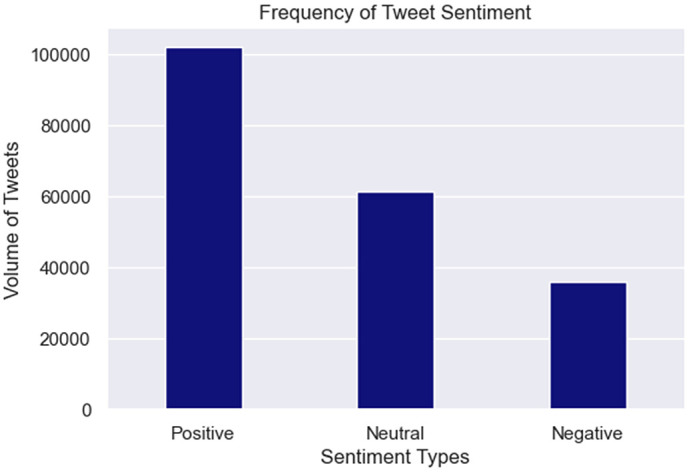
Frequency of tweet sentiment.

As visualized in Fig 3, more than half of the tweets expressed positive sentiment (51%, n = 101,878), about one-third of them expressed neutral sentiment (31%, n = 61,400), the remaining tweets (18%, n = 35946) expressed negative sentiment. Beyond these aggregate statistics, sentiment expression is more meaningful when viewed in relation to the latent topics introduced earlier. Examining this relationship helps to reveal topics that might have received disproportionately positive or disproportionately negative sentiment, thereby pinpointing specific themes that stakeholders find particularly promising or concerning. As the primary interest of this analysis is comparing positive and negative sentiments, tweets with a neutral sentiment were excluded. This reduced the data to 137,824 tweets.

Subsequently, a chi-squared test of association was conducted [48], which found a statistically significant association between the latent topics and tweet sentiment (χ^2^ (df = 12) = 1271, p < 0.01). To determine which specific combinations of latent topics and sentiment expressions are responsible for the significant association, adjusted residuals were computed, which are standardized residuals from a chi-squared test corrected for the observed counts in each cell [49]. The standardized residuals are computed as standardized differences between the observed and expected counts in each cell of the contingency table. By adjusting for the observed counts in each cell, the adjusted residuals provide a more accurate measure of the significance of the deviation between the observed and expected counts. Larger adjusted residuals indicate a stronger contribution to the chi-squared test statistics. Specifically, adjusted residuals with an absolute value of 2 or more lead to rejection of the null hypothesis [48]. The contingency table, along with the adjusted residuals, is reported in Table 4.

**Table 4:** Contingency table with residuals.

		Sentiment		Marginals (observed)
Latent topics		Negative	Positive
Topic 1 – Resources	Observed counts	2909	12884	15793
	Expected counts	4119	11674	
	Standardized residuals	−18.85	11.2	
	Adjusted residuals	−12.42	2.59	
Topic 2 – Learning assistant	Observed counts	3769	12737	16506
	Expected counts	4305	12201	
	Standardized residuals	−8.17	4.85	
	Adjusted residuals	−5.32	1.11	
Topic 3 – Educational technology	Observed counts	5031	13410	18441
	Expected counts	4810	13631	
	Standardized residuals	3.2	−1.9	
	Adjusted residuals	2.05	−0.43	
Topic 4 – Examination and assessment	Observed counts	2014	3809	5823
	Expected counts	1519	4304	
	Standardized residuals	12.71	−7.55	
	Adjusted residuals	9.02	−1.88	
Topic 5 – Productivity tool	Observed counts	9869	30793	40662
	Expected counts	10605	30057	
	Standardized residuals	−7.15	4.25	
	Adjusted residuals	−1.38	0.74	
Topic 6 – Research tool	Observed counts	2860	6673	9533
	Expected counts	2860	7046	
	Standardized residuals	7.49	−4.45	
	Adjusted residuals	4.83	−1.07	
Topic 7 – School adoption policy	Observed counts	9494	21572	31066
	Expected counts	8102	22964	
	Standardized residuals	15.46	−9.18	
	Adjusted residuals	8.8	−1.84	
Marginals (observed)		35946	101878	137824

The adjusted residuals from Table 4 indicate that *Topic 3 – Educational technology, Topic 4 – Examination and assessment, Topic 6 – Research tool,* and *Topic 7 – School adoption policy* received greater negative sentiment than would be expected by chance. In contrast, *Topic 1 – Resources* received greater positive sentiment than would be expected by chance. These findings suggest that themes related to examination, assessment, tutoring, and research attracted greater negative sentiment. This is likely due to the various risks associated with uncritical adoption of GenAI in these areas, such as accepting inaccurate or unreliable outputs, developing overreliance, and committing plagiarism [5,44]. These findings echo observations in the academic literature that poorly considered integration of GenAI into learning, research, and assessment may do more harm than good to the higher education system [43]. Conversely, stakeholders expressed significantly more positive attitudes toward the courses and resources designed to teach GenAI skills. This optimism likely stemmed from the belief that resources foster GenAI literacy, which in turn promotes responsible adoption of the technology [50].

## 6. Discussion

Owing to their remarkable proficiency in academic tasks, GenAI systems have been widely adopted in higher education, prompting extensive scholarly exploration about their opportunities and challenges [2]. Academic discussions exhibit both optimism and anxiety, highlighting the technology’s inherently dual nature [4]. The present study contributes to the literature by analyzing large-scale public discourse where relevant stakeholders are represented. The study analyzed over 230K tweets using topic modeling and sentiment analysis. The results revealed emerging themes and prevailing attitudes among stakeholders, highlighting the value of capturing stakeholders’ discourse at scale for insight into the implications of a disruptive educational technology for learning, teaching, and research.

First, the topic modeling algorithm generated seven broad topics that encapsulated stakeholders’ discourse on social media. These topics reveal stakeholders’ major points of optimism and concerns pertaining to GenAI adoption in higher education, such as learning assistance, research support, productivity enhancement, and assessment and examination [5,14]. It is notable that these themes align closely with academic discussions on the applications of GenAI in higher education. Similarly, the identified themes represented stakeholders’ prevailing attitudes from the early days of ChatGPT. Many of the concerns that later became mainstream in academic and policy circles were already being discussed by stakeholders in the early days of ChatGPT. This pattern highlights a valuable opportunity to leverage stakeholder discourse for early insight into the role of disruptive educational technologies.

Second, the topic model identified areas of interest to stakeholders that did not garner as much attention among academics, including the technology and companies behind GenAI models (Topic 3) and the resources available to develop GenAI skills (Topic 1). These themes point to two relevant developments. First, stakeholders’ social media discourse sensed, early on, the emergence of an ecosystem of technology companies, service providers, and consultants aiming to supply the tools, skills, and solutions necessary for effective integration of GenAI in higher education. Recognizing the value of this emerging ecosystem, stakeholders actively discussed its implications in their discourse, making an important connection between GenAI as an emerging technology and its role in learning and instruction. Second, stakeholders framed GenAI as an educational technology, thereby drawing attention to the importance of technical literacy to effectively utilize it for learning, instruction, and research. This characterization brought GenAI literacy to the fore of stakeholders’ discourse, where technical proficiency in using GenAI models is seen as instrumental for their effective adoption in higher education institutions.

Third, the sentiment analysis of the tweet corpus revealed a mix of GenAI optimism and anxiety among stakeholders. The optimism is captured by a large proportion of positive sentiment, demonstrating the promises of GenAI for improving student learning, supporting research, and enhancing productivity. The anxiety is captured by the smaller yet notable proportion of negative sentiment, reflecting the risks of GenAI in developing overreliance, fostering academic dishonesty, and exposing students and educators to potentially inaccurate and misleading outputs. More interestingly, the findings showed that stakeholders’ sentiment varied across the latent topics. Specifically, GenAI themes related to examination, assessment, and research elicited greater negative sentiment than would be expected by chance. So did GenAI as an educational technology. The disproportionately negative sentiment associated with these themes is likely due to increased concerns about academic integrity issues arising from the poor integration of GenAI into teaching, learning, and research. Additionally, the technology’s inherent tendency to hallucinate might have led to the fear of exposure to inaccurate or misleading outputs. In contrast, themes related to resources for learning GenAI skills—such as courses, tools, and tutorials—garnered greater positive sentiment. Resources positively resonated with stakeholders, likely because they help build the technical and ethical proficiency to responsibly adopt GenAI for academic purposes.

## 7. Future research direction

One of the benefits of big data research is its ability to reveal macro structures and patterns. Once those structures and patterns are revealed, researchers can look deeper to answer granular questions using micro-level observations. In this spirit, and given the importance of GenAI’s application as a learning assistant in the topic model, specific research questions related to this application can be formulated and examined in future research. For instance, which specific learning strategies do students use in their interaction with GenAI tools? Identifying the strategies that lead to successful learning outcomes can guide the design of more effective GenAI-based educational interventions. Likewise, future research can examine to what extent the adoption of GenAI tools contributes to students’ learning outcomes by quantifying their impact on students’ knowledge acquisition, problem-solving skills, and academic engagement.

Second, considering the importance of topics related to resources, future research might examine the structure, content, and quality of these resources. Such research can generate insight that can help to improve the design and delivery of GenAI-related courses and resources. Third, considering the importance of the technology behind GenAI and the ongoing innovation around it, future research might examine the implications of some of these ongoing innovations for the education sector. How do these innovations impact the teaching and learning process? How can students and teachers responsibly leverage advances in GenAI for more effective performance? Lastly, the ecosystem of tutors, experts, consultants, AI-powered solutions, and service providers flourishing alongside the adoption of GenAI in the education sector is relatively underexplored. Further research is needed to examine the role of this ecosystem.

## 8. Concluding remarks

This study examined stakeholders’ discourse on social media focused on the role of GenAI in higher education. By analyzing the perspectives of diverse stakeholders at scale, the study addressed a key limitation in the extant literature, wherein findings typically addressed immediate classroom concerns based on micro-level observations of students and educators. Stakeholders’ discourse on social media underscored GenAI’s transformative potential by highlighting its role in student learning, research support, and productivity enhancement. At the same time, stakeholders were acutely aware of the risks associated with uncritical GenAI adoption, including exposure to misleading content, overreliance, and academic dishonesty.

Notably, the dataset captured early public reactions to GenAI. The fact that the online discourse surfaced key implications during the introductory phase of GenAI, such as GenAI literacy, academic integrity, and responsible adoption policy, suggests that such data can help make sense of the trajectory and consequences of disruptive technologies. Such early insights can be leveraged to make sense of the implications of emerging educational technologies. Furthermore, stakeholders’ discourse proved highly relevant in identifying key GenAI adoption themes in higher education, such as learning, research, productivity, assessment, and adoption policy. This finding suggests that educators and policymakers could use social media discourse as an input for policy-making. For instance, based on current findings, policymakers can take steps to develop GenAI literacy among students and faculty, which should encourage responsible adoption for learning and research. This can be pursued in various ways, for instance, by integrating GenAI literacy content into existing courses, designing new GenAI-focused courses, or through short-term training programs and seminars. Similarly, academic integrity can be stressed as a key requirement of using GenAI tools for academic tasks. This can be formalized through course policies and research guidelines. To encourage adherence, the consequences of irresponsible use on student learning, research integrity, and assessment outcomes can be spelled out and clearly communicated.

To conclude, this study demonstrates the promise of analyzing social media data to generate early insight about disruptive educational technologies. However, such analysis cannot substitute for an institutional procedure, where contextually relevant input is gathered from internal and external sources and policies are devised through established channels. Social media analytics is best utilized as a complementary rather than a substitute for institutional procedures.

## Supporting information

S1 Dataset Topic modeling and sentiment analysis dataset. (XLSX)
